# Assessment of Chronic Malnutrition and Its Correlation With Oral Health Status in Children Aged Three to Six Years in Jabalpur District, India: An Epidemiological Study

**DOI:** 10.7759/cureus.67838

**Published:** 2024-08-26

**Authors:** Aishwarya Jethi, Debapriya Pradhan, Saurabh Tiwari, Ankit Dhimole, Nikita Saini, Ankita Yadav, Namrata Jain, Delphina Michael Kapoor

**Affiliations:** 1 Department of Pedodontics and Preventive Dentistry, Hitkarini Dental College and Hospital, Jabalpur, IND; 2 Department of Oral Medicine and Radiology, Hitkarini Dental College and Hospital, Jabalpur, IND

**Keywords:** dental enamel hypoplasia, dental caries, salivary buffering capacity, salivary ph, salivary flow rate, weight-for-height z-scores

## Abstract

Objective: This study aims to examine the correlation of chronic malnutrition with the oral health status of children aged three to six years.

Methods: A total of 400 children were selected and divided into four groups based on z-scores. For evaluation of oral health status, teeth were examined for dental caries using the decayed, missing, and filled permanent teeth (DMFT) scores. Salivary samples were collected to measure salivary flow rate (SFR), pH, and salivary buffering capacity (SBC). Teeth were also visualized to detect the presence or absence of enamel hypoplasia. The data obtained was statistically analyzed (p<0.05).

Results: A total of 400 children participated and were also categorized into mild, moderate, and severe obesity according to the z-scores. The mean DMFT scores among adequately nourished children were 2.4086; with severely malnourished and severely obese children, the mean values were found to be 1.0652 and 1.4286, respectively. The mean SFR among children with adequate nutrition was 1.0366, and the mean flow rate among children with severe malnutrition and obesity was 0.5348 and 0.4036, respectively. The mean salivary pH among children with adequate nutrition was 7.1295, and the mean values for participants with severe malnutrition and severe obesity were found to be 6.4772 and 7.6521, respectively. The mean SBC among children with adequate nutrition was found to be 4.4861; for severely malnourished children and those with severe obesity, the values were 3.2472 and 2.8332, respectively. There was an absence of enamel hypoplasia in children with adequate nutrition, whereas a total of five participants with severe malnutrition and three children with severe obesity were found to have hypoplastic lesions, respectively.

Conclusion: Malnutrition exerts a negative impact on the overall oral health of children. It is critical to diagnose the effects of malnutrition on children's oral environments in order to provide appropriate treatment and enhance their quality of life.

## Introduction

Ideal dental health is influenced by a balanced diet that can supply enough nutrition. Both poor nutrition and the use of particular foods have an impact on the oral cavity. As we all know, dental caries is still a significant public health issue on a global scale [1]. It has been observed that intrauterine, prenatal, and postnatal conditions can all contribute to protein-calorie deficiency [2]. In both industrialized and developing nations, populations that are nutritionally undernourished have higher rates of childhood caries [3]. Malnutrition is an energy or protein deficit state that affects the body's ability to operate and can have detrimental clinical effects if left untreated [4]. The majority of child fatalities have been linked to undernutrition in large-scale worldwide research, with substantial relative mortality rates for severe malnutrition. Malnutrition is frequently a result of illness, long-term diseases, trauma, burns, or surgery in industrialized countries [5]. Children in India are malnourished due to factors such as poverty, large families, poor maternal health, harmful cultural practices, environmental degradation, a lack of education, gender inequality, and unaffordable medical care. Malnourished children of growing ages may exhibit behavioral changes such as irritability, apathy, reduced social responsibility, anxiety, inattention, stunted growth, poor school performance, and reduced intellectual achievement. However, malnutrition is also known to cause high morbidity and mortality, and when considering its effects on the oral cavity, malnutrition has been shown to have both initial and post-initial effects [2]. According to the recent National Family Health Survey (NFHS-3), child mortality due to malnutrition in India is 45 out of 1000 live births [6]. Dental caries increases in developing nations along with development, and children are more at risk for disease disadvantage. The need to precisely identify high caries risk groups, to start prevention at a young age, and to investigate the impact of early intervention in children on general and dental health with both population and high-risk approaches is becoming increasingly apparent globally. It is important to consider social and cultural aspects of child development, such as family stress, health service use and access, nutrition (including use of sugar and access to fluoride), oral microflora activity and composition, and awareness of behavioral and biological effects on health. Key risk factors and the emergence of dental caries in children have been examined cross-sectionally and, in some cases, longitudinally in published research. However, little is known about the connections between molecular effects and psychosocial effects in the context of developing nations, particularly within and between children from underprivileged, disadvantaged backgrounds [7]. Oral tissues need nutrition for growth, maintenance, and development, and dental concerns can affect dietary preferences and eventually nutritional status.

Nutritional deficiencies can have an impact on tooth morphology and size prior to eruption, as well as enamel maturation and composition. A significant proportion of primary teeth are carious as a result of undernutrition in children, which further worsens the carcinogenicity of dietary sugars. Overall, it is abundantly obvious that undernutrition is a result of tooth decay and early tooth loss [8]. It is well known that dental caries is an infectious illness that can be prevented and that food has a significant impact on it. The major factors in the disease's etiology include cariogenic bacteria, fermentable carbohydrates, a vulnerable tooth, and the host, as well as these factors. However, in infants and young children, bacterial flora and host defense systems are still developing, tooth surfaces are just emerging and may have hypoplastic defects, and their parents must navigate the dietary transition through breast/bottle feeding, first solids, and childhood tastes. As a result, it is believed that there may be specific risk factors for caries in these age groups [7]. A complex biological fluid, saliva contains components brought from the blood as well as enzymes, hormones, antibacterial agents, and electrolytes. The physiological status of the organism, including hormonal, nutritional, and metabolic abnormalities, is thus reflected by it. Saliva plays a crucial role in the mechanism of dental caries, and raising the salivary flow rate (SFR) may improve protection against the emergence of caries lesions [9]. To maintain good oral health, saliva is crucial. In the oral cavity, it aids in preserving homeostasis. Buffering and neutralizing the acids created by cariogenic organisms reduces the likelihood of tooth caries. Protein-energy malnutrition has been linked to hypofunctioning of the salivary glands, which lowers the SFR and buffering capacity and lowers the oral cavity's ability to fight infections. Additionally, a lack of vitamins and other minerals might alter the composition and volume of saliva, which could compromise its protective properties in the oral cavity [10]. In addition to the above-mentioned causes, enamel hypoplasia, a built-in flaw in teeth that can be caused by starvation, raises the susceptibility to caries [11]. Due to the disruption of ameloblastic activity during the secretory phase of amelogenesis, enamel hypoplasia is linked to malnutrition. The size of the defect and the translucency of the partially produced enamel depend on how severe the injury is. Ameloblastic activity is likely to be disturbed by both moderate and severe malnutrition [12]. It has been noted that diet is a common risk factor for dental health status and nutrition and that these factors are likely to affect quality of life. Additionally, these characteristics are more likely to worsen among underprivileged populations and less developed nations where access to and affordability of dental treatment are more limited [13]. In order to assess the impact of chronic malnutrition on the oral health status of children between the ages of 3 and 6, the current study analyzed caries experiences according to the severity of malnutrition to ascertain whether there was a relationship between nutritional status and both the quality and quantity of saliva, as well as a correlation between chronic malnutrition and enamel hypoplasia.

## Materials and methods

The present observational, analytical, cross-sectional study was carried out in various government schools in the district in collaboration with our institute’s biochemistry lab. The study was approved by the Institutional Ethics Committee of Hitkarini Dental College and Hospital (approval number: 22/753/N). This study was conducted to evaluate the effect of chronic malnutrition on caries prevalence, salivary status, and the prevalence of enamel hypoplasia. Children falling under the age group of three to six years of age, whose parents have signed informed consent, were included in the study. Children without any systemic disease or any disability limitations were included in the study. Children with scanty salivary flow (<0.1 ml/min) were excluded from the study.

Four hundred children were selected and allocated into four groups according to the z-scores for nutritional status (WHO 2009) taken for the evaluation of dental caries, saliva, and enamel hypoplasia (Table 1).

**Table 1:** Allocation of subjects according to the WHO (2009) standard deviation (z-score) with nutritional status WHO: World Health Organization

Groups	Standard deviation (z-score) with nutritional status	No. of children
Group I	+1.5 to -1.5 (adequate nutrition)	n = 100
Group II	≥-1.5 (mild malnutrition)	n = 100
Group III	≥-2 (moderate malnutrition)	n = 100
Group IV	≥-3 (severe malnutrition)	n = 100
	Total number of children	N = 400

The children were asked to weigh on a calibrated electronic scale (capacity: 150 kg; precision: 100 g) barefoot and wearing light clothing in the presence of the mother or caregiver. A non-flexible metric tape measuring up to two meters in length with 0.1-centimeter accuracy was used to measure height. Measurements were made twice, and the mean was used for the calculation of height for age, weight for height, and weight for age. The guidelines of the WHO (2009) were the references for the evaluation of nutritional status, as previously mentioned in the table.

The dental examination was performed in a dental chair under conventional light. Prior to the examinations, the children’s teeth were cleaned with a toothbrush and toothpaste. A flat mouth mirror, tongue depressor, and gauze (to clean and dry the teeth) were used during the examinations to enable the visualization of areas with non-cavitated lesions (white spots - areas of demineralization of the enamel with the loss of translucence-opaque white lesions without cavitation). No exploratory probes were used to avoid the transference of microorganisms from one surface to another and the possibility of damaging the demineralized surface of the enamel. Dental caries experience was recorded using the decayed, missing, and filled permanent teeth (DMFT) index, which was employed following the guidelines of WHO (2009) to establish the prevalence and severity of caries. Samples of unstimulated saliva were collected from the participants for five minutes. The quantity of saliva was measured after five minutes in order to calculate salivary flow. Collections were performed between 9 and 11 a.m., during which the child did not eat anything. The volume of the saliva was measured. After the measurement of salivary flow, the initial pH was measured with a potable pH meter. For the determination of salivary buffering capacity (SBC), an aliquot of 1 ml was transferred to a test tube with 3 ml of hydrochloride acid (HCl 5 mM) for titration. The mixture was vigorously shaken, centrifuged for one minute, and allowed to stand for 10 minutes, and then the final pH was measured. Similar to the dental caries evaluation, enamel hypoplasia was also examined. A mouth mirror and a conventional light were used to visualize enamel hypoplasia. Teeth were scrubbed with gauze sponges to facilitate detecting the hypoplastic lesion.

Data was analyzed using SPSS Statistics version 26.0 (IBM Corp., Released 2019; IBM SPSS Statistics for Windows, Version 26.0; Armonk, NY: IBM Corp.). The data was entered in Microsoft Excel 2016 for Windows (Microsoft Corporation, Redmond, WA, USA). The frequencies and percentages of gender were calculated. The data was categorical, and Pearson’s Chi-square test was applied for data analysis for the presence and absence of enamel hypoplasia and the distribution of participants among different nutritional statuses. Mean values for height, weight, SFR, SBC, salivary pH, and DMFT score were calculated using an ANOVA test among the nutritional status categories. A p-value of <0.05 was considered statistically significant.

## Results

Overall, 400 children with a mean age of three to six years of age were recruited; 239 (59.8%) were males and 161 (40.3%) were females (Table 2).

**Table 2:** Gender distribution of participants

Gender	N	%
Males	239	59.8%
Females	161	40.3%

**Figure 1:**
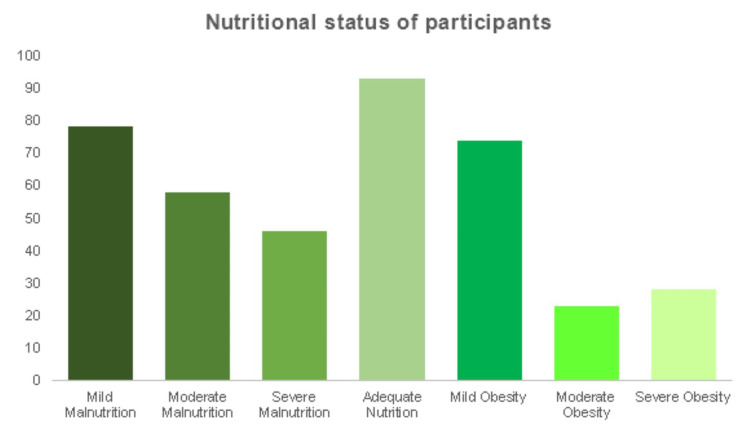
Distribution of participants based on nutritional status

Figure 1 shows the distribution of participants based on nutritional status. Apart from different types of malnutrition, obese children were also found, so they were also included in the study. Ninety-three participants fell into the category of adequate nutrition, 74 participants belonged to mild obesity, 23 participants had moderate obesity, 28 participants fell within the category of severe obesity, 78 participants reported mild malnutrition, 58 participants fell under moderate malnutrition, and 46 participants had severe malnutrition.

Table 3 shows the comparison of mean values of SFR among the different nutritional status categories. The mean SFR among participants with adequate nutrition was 1.0366; the mean SFR among participants with severe malnutrition and severe obesity was found to be 0.5348 and 0.4036, respectively. However, significant differences in SFR were found among nutritional status categories.

**Table 3:** Correlation between SFR and nutritional status SFR: salivary flow rate

Categories	N	Mean	Std. deviation	P-value
Mild malnutrition	78	0.5218	0.12342	0.000
Moderate malnutrition	58	0.4897	0.27764	
Severe malnutrition	46	0.5348	0.44831	
Adequate nutrition	93	1.0366	0.49184	
Mild obesity	74	1.3108	0.80420	
Moderate obesity	23	0.3435	0.09921	
Severe obesity	28	0.4036	0.13189	

Table 4 shows the comparison of mean values of SBC among the different nutritional status categories. The mean SBC among participants with adequate nutrition was 4.4861, the mean SFR among participants with severe malnutrition was 3.4272, and the SBC among participants with severe obesity was 2.8332. There were significant differences in SBC among nutritional status categories.

**Table 4:** Correlation between SBC and nutritional status SBC: salivary buffering capacity

Categories	N	Mean	Std. deviation	P-value
Mild malnutrition	78	3.5151	0.55251	0.000
Moderate malnutrition	58	3.1534	1.08732	
Severe malnutrition	46	3.2472	1.20672	
Adequate nutrition	93	4.4861	0.82807	
Mild obesity	74	4.7608	1.62583	
Moderate obesity	23	2.5665	0.73097	
Severe obesity	28	2.8332	0.82059	

Table 5 shows the comparison of mean values of salivary pH among the different nutritional status categories. The mean salivary pH among participants with adequate nutrition was 7.1295, the mean salivary pH among participants with severe malnutrition was 6.4772, and the SBC among participants with severe obesity was 7.6521. The highest salivary pH was seen among participants with severe obesity, and the lowest salivary pH was seen in children with severe malnutrition. There were significant differences in salivary pH among different nutritional status categories.

**Table 5:** Correlation between salivary pH and nutritional status

Categories	N	Mean	Std. deviation	P-value
Mild malnutrition	78	7.4597	0.93057	0.000
Moderate malnutrition	58	6.9279	1.01386	
Severe malnutrition	46	6.4772	1.05176	
Adequate nutrition	93	7.1295	1.10963	
Mild obesity	74	7.2189	1.00372	
Moderate obesity	23	6.8065	1.06730	
Severe obesity	28	7.6521	1.08756	

Table 6 shows the comparison of the mean values of DMFT scores among the different nutritional status categories. The mean DMFT score among participants with adequate nutrition was 2.4086, the mean DMFT score among participants with severe malnutrition was 1.0652, and the mean DMFT score among children with severe obesity was 1.4286. There were significant differences in mean DMFT scores among nutritional status categories.

**Table 6:** Correlation between DMFT and nutritional status DMFT: decayed, missing, and filled permanent teeth

Categories	N	Mean	Std. deviation	P-value
Mild malnutrition	78	1.4359	2.65110	0.000
Moderate malnutrition	58	1.3621	1.97082	
Severe malnutrition	46	1.0652	1.85475	
Adequate nutrition	93	2.4086	3.37586	
Mild obesity	74	1.8514	2.29760	
Moderate obesity	23	2.9565	2.43979	
Severe obesity	28	1.4286	2.65872	

Table 7 shows the comparison of the distribution of participants with enamel hypoplasia. A total of 17 participants had enamel hypoplasia among the different nutritional status categories. The highest prevalence was found in children with severe malnutrition, with a total of five participants. A total of three participants with severe obesity were found to have enamel hypoplasia. No participants were seen to have enamel hypoplasia in the category of adequate nutrition.

**Table 7:** Presence of enamel hypoplasia found in children with different levels of nutritional status

Categories	N	Yes	No
Mild malnutrition	78	2	76
Moderate malnutrition	58	2	56
Severe malnutrition	46	5	41
Adequate nutrition	93	0	93
Mild obesity	74	4	70
Moderate obesity	23	1	22
Severe obesity	28	3	25

## Discussion

The study offers vital new information about the complex connection between nutritional deficiencies and oral health in children aged three to six years. This study is consistent with a broader body of literature that highlights the detrimental effects of malnutrition on oral health and demonstrates a cycle of poor health outcomes that is frequently exacerbated by socioeconomic factors, dietary practices, and limited access to healthcare. Chronic malnutrition, a persistent problem in many parts of India, directly affects children's growth and development [14]. Children in Jabalpur had a high prevalence of both wasting and stunting, which is consistent with findings from other areas facing comparable socioeconomic difficulties. This study also examined the severity of childhood obesity. For example, Bhutta et al. (2008) reported that chronic malnutrition contributes to delayed growth and development in children, which can manifest in various health issues, including poor oral health [15]. Poor oral health, particularly dental caries and periodontal diseases, is prevalent among malnourished children [16]. The study highlighted a significant correlation between malnutrition and dental caries, consistent with findings from Moynihan (2005) and Petersen and Ogawa (2012), who also identified a higher incidence of dental caries in malnourished populations [17,18]. This correlation can be attributed to several factors, including weakened immune systems and delayed dental development, which increase susceptibility to oral infections and diseases. Similar to dietary patterns seen in other low-income environments, a large number of children in Jabalpur had diets high in sugar and low in vital nutrients. Studies by Kalsbeek and Verrips (1994) and Marshall et al. (2003) showed that diets rich in sugary snacks and beverages contribute significantly to dental caries, especially in the absence of adequate oral hygiene practices [19,20]. Furthermore, oral health is made worse by inadequate nutrition brought on by a lack of access to a balanced diet. Additionally, the socioeconomic environment has a major impact on oral health and malnutrition. Children from poorer socioeconomic backgrounds are more likely to experience food insecurity, which increases their risk of malnutrition, and they frequently lack access to dental care. The study also revealed that children hailing from impoverished families had elevated incidences of dental diseases and malnourishment, underscoring the necessity for policies that tackle these socioeconomic disparities.

The results of the study on children's oral hygiene habits in Jabalpur are consistent with a larger pattern seen in comparable contexts. Lack of knowledge about good oral hygiene among many caregivers resulted in poor habits like infrequent toothbrushing and irregular dental checkups. Improving oral hygiene practices through community-based education programs could significantly reduce the incidence of dental caries and improve overall health outcomes, as suggested by Vanobbergen et al. (2001) and Pine and Harris (2007) [21,22]. Oral health promotion programs, in conjunction with nutritional education, have demonstrated potential in terms of enhancing health outcomes. Helderman and Benzian (2006) and Petersen and Kwan (2004) recommended community-based programs that train health workers to provide both dietary advice and basic oral care [23,24]. The study indicates that in order to achieve meaningful improvements in health, it is imperative to break the cycle of malnutrition and poor oral health, which can be facilitated by such integrated approaches. Addressing the combined burden of malnutrition and poor oral health requires effective policy measures. Programs that involve local authorities, schools, and community health workers can effectively promote oral and nutritional health. The study highlights the need for mobile dental clinics and community nutrition programs to address the issue of healthcare access in underserved areas. In order to address nutritional deficiencies and oral health concerns, healthcare professionals are needed. It is recommended that pediatricians, dentists, and nutritionists work together to offer children comprehensive health care. To detect and treat problems early, nutritional evaluations and oral health screenings should be part of routine health checkups. Training healthcare providers to recognize signs of malnutrition and oral diseases can improve early intervention and treatment, a recommendation supported by Moynihan and Petersen (2004) [25]. Prevention of the long-term effects of malnutrition and poor oral health requires early intervention. As noted by Bhutta et al. (2008) and Dewey and Adu-Afarwuah (2008), programs aimed at pregnant women and young children can guarantee that children receive essential nutrients and develop good oral hygiene practices from an early age [15,26]. Addressing malnutrition and oral health issues in resource-constrained settings like Jabalpur presents several challenges. Attempts to enhance health outcomes may be hampered by cultural beliefs, socioeconomic inequality, and limited access to healthcare. As discussed by Petersen and Ogawa (2012), overcoming these obstacles calls for a multifaceted strategy that includes community involvement, policy changes, and ongoing funding for health programs [18]. The research underscores the necessity for additional investigation to fully comprehend the intricate correlation between oral health and malnourishment. Studies with a longitudinal design are required to investigate the long-term impacts of integrated interventions and prove causation. In order to address deficiencies, research should concentrate on identifying particular nutrients that are important for oral health and creating focused interventions.

The study has limitations, even though it offers insightful information. The cross-sectional design limits the ability to establish causality between chronic malnutrition and poor oral health. Additionally, the reliance on caregiver-reported data may introduce reporting bias, particularly regarding dietary habits and oral hygiene practices. Future studies should explore the underlying mechanisms linking malnutrition and oral health in greater detail. Studies investigating the role of specific nutrients, such as vitamins and minerals, in oral health could provide more targeted intervention strategies. Furthermore, studies on the efficiency of integrated health interventions in lowering oral diseases and malnutrition would be beneficial for developing public health policies and initiatives.

This epidemiological study underscores the significant correlation between chronic malnutrition and poor oral health among children aged three to six years in Jabalpur. The high prevalence of both conditions highlights the need for comprehensive public health interventions that address the multifaceted nature of child health. By integrating nutritional and oral health initiatives, it is possible to improve the well-being of children in resource-constrained settings, ultimately reducing the burden of malnutrition and oral diseases.

## Conclusions

The findings of this epidemiological study underscore the critical link between chronic malnutrition and oral health status among children aged three to six years in Jabalpur. The study revealed a significant prevalence of chronic malnutrition within this population, with a notable proportion of children experiencing stunting, wasting, and being underweight. Furthermore, the research identified a strong correlation between chronic malnutrition and poor oral health outcomes, including higher rates of dental caries and suboptimal oral hygiene practices among malnourished children. These findings highlight the urgent need for integrated public health interventions that address both nutritional deficiencies and oral health disparities among young children in the region. Moving forward, targeted efforts are necessary to develop and implement comprehensive health programs that promote proper nutrition and oral hygiene practices from an early age. Such interventions should involve collaboration between healthcare providers, policymakers, educators, and community stakeholders to ensure holistic support for children's health and well-being. By addressing the intertwined challenges of chronic malnutrition and poor oral health, we can enhance the quality of life for children in Jabalpur and empower them to thrive physically, mentally, and socially as they grow and develop.
